# Predictors of COVID-19 Vaccination Intention and Behavior Among Young People in a European Union Country With Low COVID-19 Vaccination Rates: Cross-Sectional Study

**DOI:** 10.2196/64653

**Published:** 2025-02-21

**Authors:** Sara Atanasova, Tanja Kamin, Nina Perger

**Affiliations:** 1 Faculty of Social Sciences University of Ljubljana Ljubljana Slovenia

**Keywords:** vaccine uptake, young people, COVID-19 vaccine, health belief model, theory of planned behavior

## Abstract

**Background:**

Vaccination against COVID-19 is a critical measure for managing the pandemic and achieving herd immunity. In 2021, Slovenia had a significantly lower COVID-19 vaccination rate compared to the average rate in the European Union, with individuals aged younger than 37 years showing the highest hesitancy. Previous studies primarily explored vaccination willingness before vaccines were available to young people, leaving a gap in understanding the factors influencing vaccination behavior and differences within the population of young people.

**Objective:**

This study aimed to investigate a wide set of predictors influencing COVID-19 vaccination intention and behavior among young people in Slovenia. Specifically, we aimed to compare vaccinated and unvaccinated young people, further categorizing the unvaccinated group into those who were hesitant, those who intended to vaccinate in the near future, and those who refused vaccination.

**Methods:**

An integrated model, based on the health belief model and theory of planned behavior, was developed, and it included additional contextual factors (such as trust in science, trust in vaccines, conspiracy theory tendencies, etc) and health-related and sociodemographic characteristics. Data were collected in August 2021 via the online access survey panel JazVem (Valicon), targeting individuals aged 15-30 years in Slovenia. Quotas ensured that the sample (n=507) was quasi-representative according to age, gender, education, and region. Bivariate analyses and multinomial logistic regression were performed to explore the determinants of vaccination intention and behavior.

**Results:**

Among respondents, 45.8% (232/507) were vaccinated, 30.0% (152/507) refused vaccination, 12.4% (63/507) were hesitant, and 11.8% (60/507) intended to undergo vaccination in the near future. Vaccinated individuals were predominantly aged 23-26 years, had higher education, and reported above-average material status. Refusers were more common among the youngest (15-18 years) and oldest (27-30 years) groups, had lower education, and showed higher conspiracy theory tendencies. Multinomial regression analysis revealed that unvaccinated respondents who perceived greater COVID-19–related health consequences were more likely to delay vaccination (adjusted odds ratio [aOR] 2.0, 95% CI 1.2-3.3) or exhibit hesitancy (aOR 1.9, 95% CI 1.1-3.2) compared with vaccinated respondents. Subjective norms were less influential among hesitant individuals (aOR 0.4, 95% CI 0.2-0.7) and refusers (aOR 0.3, 95% CI 0.2-0.7) than among vaccinated individuals. Self-efficacy in managing health problems was less evident among those who delayed vaccination to the near future (aOR 0.5, 95% CI 0.3-0.9) than among vaccinated individuals.

**Conclusions:**

This study underscores the complexity of vaccination intentions and behaviors among young people, emphasizing the necessity for public health strategies promoting vaccination to be tailored to the specific reasons for nonvaccination within different subgroups. Interventions aimed at addressing vaccine hesitancy and delays should particularly focus on individuals with lower education and material disadvantages. By fostering trust and enhancing self-efficacy, these interventions could more effectively promote vaccine uptake.

## Introduction

The rapid development of COVID-19 vaccines is considered as a pandemic success story, and vaccination against COVID-19 is considered as one of the most effective preventive measures [1,2]. The herd immunity threshold for SARS-CoV-2 has been assumed to be between 50% and 67%, and the administration of SARS-CoV-2 vaccines was suggested to be an important strategy to reach this threshold [3]. It has been suggested that around 75% to 90% of the population needs to be vaccinated against COVID-19 to successfully control the pandemic [4]. Slovenia lagged far behind this goal; it was among the countries with the lowest vaccination rate against COVID-19 in the European Union (EU) since vaccination against COVID-19 became available to citizens [5]. By the end of 2021, the 27 EU member states had vaccinated an average of 77% of their population aged 18 years or older. However, Slovenia reported a vaccination completion rate of only 65.6% among adults [6].

Individuals showing the most hesitancy regarding COVID-19 vaccination in Slovenia were those younger than 37 years [7]. This is consistent with the findings of previous studies globally that among adults, younger age groups had a lower willingness to be vaccinated against COVID-19 [8-15]. Vaccination against COVID-19 among those most likely to get infected and to transmit the virus, such as young people in school settings, was identified as a crucial strategy for reducing ongoing transmission and preventing outbreaks [16]. This approach was considered essential for establishing herd immunity; protecting elderly people and immunocompromised individuals from severe outcomes, hospitalization, and death [17]; and ensuring the continued functioning of the workforce [16].

The vaccination behavior of young people is complex, as highlighted by a systematic review of qualitative studies on adolescents’ understanding and attitudes toward vaccines [18]. It has been suggested that health literacy plays a crucial role in adolescents’ comprehension of the benefits and risks of vaccination. However, other factors, such as peer influence, parental guidance, trust in health care providers, and misconceptions about the vaccine’s safety and efficacy, also significantly influence vaccine uptake among adolescents [18]. Given the unprecedented speed at which COVID-19 vaccines were developed compared to historical vaccine development and testing timelines, a higher degree of hesitancy toward COVID-19 vaccination was somewhat anticipated. A study examining the factors that discourage individuals from participating in vaccine trial registries or clinical trials identified primary concerns related to the safety of novel vaccines and a lack of trust in those involved in vaccine development [19].

Similar findings were obtained for vaccination behavior related to COVID-19, with attitude toward vaccination, trust in health staff and scientists, time of information, and conspiracy beliefs about COVID-19 being identified as the best predictors of the intention of vaccination among young adults in Spain at the beginning of the vaccination campaign [20]. Chaufan [21], for example, questioned the prevailing risk-benefit analysis with regard to younger people. If vaccine uptake significantly reduces health risks in the general population, the younger population has a much lower risk of COVID-19–related complications. Given their lower risk of COVID-19–related health complications, the reluctance to vaccinate in this population group, which is often seen as unreasonable, may seem understandable [22]. Their perceived benefits of vaccination seem to be more collectively beneficial, while the risks associated with vaccination are perceived to be individual. This vaccination was new, and there was no broad-based data available on the efficacy and immunogenicity of the vaccination in the young population at the beginning of the vaccination campaign against COVID-19 [23].

Another scoping review suggested that the primary factors of young people’s acceptance of the COVID-19 vaccine include the desire to protect themselves and close family members or friends, fear of infection, professional recommendations, and employer obligations [24]. On the other hand, the primary hesitancy factors include concerns about the safety of the vaccine and its side effects, effectiveness, and efficacy, as well as a lack of trust in the pharmaceutical industry and government, conspiracies, and favoring natural immunity [24]. The same study also suggested the need for additional research into COVID-19 vaccine–related decision-making dynamics for specific adolescent and youth population age ranges to better understand how vaccination-related behavior is influenced by environmental or social factors as well as personal health and susceptibility [24].

The significant affective distress experienced by the younger population due to COVID-19–related protective measures, particularly isolation and social distancing [25], may substantially influence both their willingness to receive the vaccination, as suggested by a study [26], and their actual uptake of COVID-19 vaccination. This is especially pertinent if vaccination is perceived as a means to achieve greater mobility and fewer restrictions on in-person social interactions. Negative emotions (affective responses) associated with recommended protective behaviors may impact core beliefs (cognitive responses) and undermine evidence-based reasoning, thereby diminishing the perceived importance of these behaviors in decision-making processes (behavioral responses) [25]. These findings highlight that vaccination intention and behavior depend on several contextual factors. Most studies conducted prior to our research primarily focused on investigating the willingness to receive COVID-19 vaccination at a time when vaccines were not yet available to young people. Furthermore, studies involving the youth have often relied on opportunistic samples, predominantly drawn from student populations [27-30]. Such approaches have resulted in narrow and unrepresentative samples that fail to adequately reflect the broader category of young people. Our study aims to address these limitations.

The main aim of this study is to investigate a wide set of predictors influencing COVID-19 vaccination intention and behavior among young people in Slovenia. Specifically, based on a quasi-representative sample of young people in Slovenia, this study aims to gain a deeper understanding of the cognitive, affective, and behavioral determinants of vaccine uptake within this population. It examines factors related to COVID-19 vaccination intention and behavior, including sociodemographic and health-related variables, as well as other predictors identified in previous studies and theoretical frameworks as significant in influencing vaccination behavior [14,24]. The study adopts and further develops the model by Shmueli [31], incorporating factors from 2 behavioral models (health belief model [HBM] and theory of planned behavior [TPB]) along with additional social factors such as trust in science and tendency toward conspiracy theories. These factors have been shown to be strong predictors of the willingness to receive the COVID-19 vaccine in the adult population [32]. Building on this integrated model, the study contributes by comparing vaccinated and unvaccinated young people and further categorizing the unvaccinated group into those who are hesitant, those who intend to vaccinate, and those who refuse vaccination. Our goal is to enhance the body of research that explores the decision-making processes within distinct groups of young people. The study seeks to better understand and inform public health strategies for increasing vaccination rates for COVID-19 and future viral outbreaks among young people.

## Methods

### Framework of the Study

**Figure 1:**
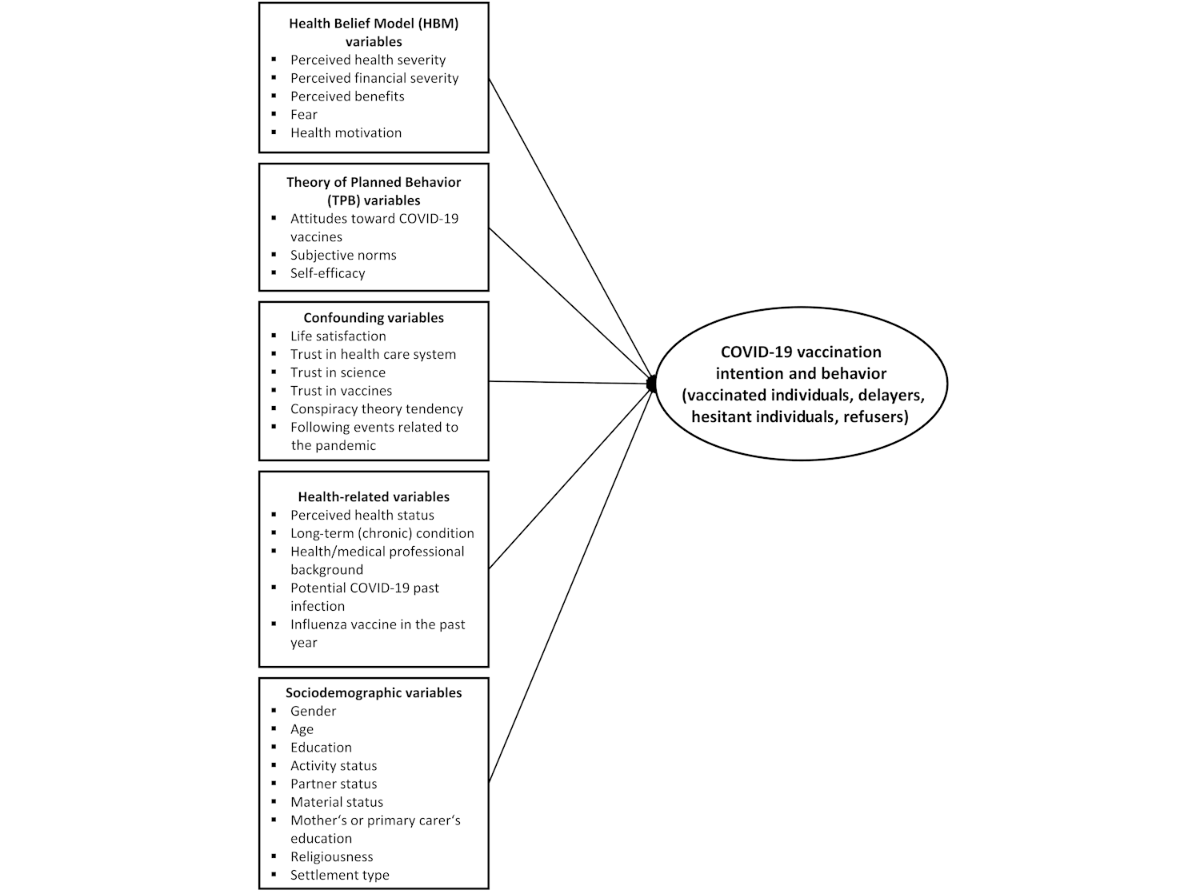
Framework of the predictors of COVID-19 vaccination intention and behavior.

Based on the study by Schmueli [31], we developed an integrated model that integrates the constructs of 2 prominent health behavior theories (HBM and TPB) and includes a number of other contextual factors (eg, trust in science, trust in vaccines, conspiracy theory tendencies, etc) and health-related and sociodemographic characteristics of the studied population to identify the predictors of COVID-19 vaccination intention and behavior among young people in Slovenia (Figure 1). The HBM, which has generated a great deal of research interest in various health-related behaviors to date, is based on a set of core beliefs (cognitive factors) regarding risk susceptibility, risk severity, benefits, and barriers [33]. According to the widely tested TPB [34,35], the underlying behavioral intention as a direct antecedent of behavior is determined by the attitude toward the behavior, subjective norms, and perceived behavioral control. Both models aim to predict behavior, and in our case, we target COVID-19 vaccination–related behavior.

### Study Design

The data for this cross-sectional study were collected using an online survey questionnaire administered by the online survey panel JazVem (Valicon, a Slovenian marketing research company) [36]. Existing members of the JazVem online survey panel were contacted via email to participate in the survey. Members voluntarily sign up with the JazVem online survey panel, agree with the terms and privacy conditions of the online survey panel, sign participation consent, provide baseline information, and regularly receive emails inviting them to take part in different research studies. For participation in JazVem surveys, panelists receive small incentives in the form of points, which can be redeemed for modest rewards. Online panels operate under rigorous recruitment and quality assurance protocols to ensure participants can enroll only once, avoid overexposure to surveys, and maintain high levels of engagement. More information about the study design can be found in the CHERRIES (Checklist for Reporting Results of Internet e-Surveys) in Multimedia Appendix 1.

### Data Collection

The online questionnaire used in this study was developed by the authors and included 79 questions in the Slovene language, which took around 15 to 20 minutes to answer. The questionnaire covered questions about the perception of COVID-19–related protective measures, questions regarding behavior for vaccination against COVID-19, questions related to HBM and TPB constructs, questions about trust in science and vaccines, questions about the respondent’s health, and questions related to sociodemographic characteristics. The questionnaire and measurement instruments were evaluated for content validity by 8 experts from the fields of public health, health communication, sociology, communication studies, statistics, social science methodology, and psychology.

Data were collected between August 11, 2021, and August 17, 2021. Eligible participants were aged 15 to 30 years and resided in Slovenia. Quotas were used to achieve a quasi-representative sample of the general population in Slovenia according to age, gender, education, and region based on data from the Statistical Office of the Republic of Slovenia. Based on quota sampling, 1197 JazVem panelists were invited to participate in the online survey. Among these, 568 clicked on the link for the online survey and 555 viewed the introduction page of the survey. Of the 568 respondents, 525 fully completed the survey, with a completion rate of 92.4% (525/568). After the data screening and cleaning procedures, the final sample comprised 507 respondents. The data were weighted to match the general population distribution according to age, gender, education, and region. More information about the data collection can be found in the CHERRIES in Multimedia Appendix 1.

### Measurement Instruments

#### Vaccine Uptake

The dependent variable vaccine uptake was measured with the following three questions: (1) “Please tell us about your decision regarding the COVID-19 vaccination. Have you been vaccinated against COVID-19?” (2) “Do you intend to get vaccinated against COVID-19 in the next 30 days?” and (3) “Do you intend to get vaccinated against COVID-19 in the next 6 months?” The classification and naming of unvaccinated respondents was guided by the Stages of Change model [37]. This model posits several behavioral stages preceding the desired recommended behavior, which in our case was vaccination behavior: (1) Precontemplation: individuals in this stage are not considering vaccination; (2) Contemplation: individuals in this stage are considering vaccinating in the next 6 months, and according to the theory, they are considering the recommended behavior but still weighing the pros and cons; and (3) Preparation: individuals in this stage intend to get vaccinated in the next 30 days, and according to the theory, they may have taken some initial steps, such as seeking information or planning an appointment. Based on the respondents’ answers, we computed vaccine uptake variables and divided the respondents into 4 categories as follows: respondents who reported being vaccinated were categorized as “vaccinated respondents;” respondents who reported not being vaccinated but intended to get vaccinated in the next 30 days were categorized as “delayers;” respondents who reported not being vaccinated but intended to get vaccinated in the next 6 months were categorized as “hesitant respondents;” and respondents who reported not being vaccinated and who did not intend to get vaccinated in the next 30 days or 6 months were categorized as “refusers.”

The independent variables (ie, predictors of vaccine uptake among young people) were arranged into 5 groups: HBM variables, TPB variables, confounding variables, health-related variables, and sociodemographic variables.

#### HBM Variables

Perceived health severity was measured with an item adapted from Shmueli [31], with respondents asked to assess on a 5-point Likert scale (1 [insignificant consequences] to 5 [very serious consequences]) the possible health consequences in the case of COVID-19 infection.

Perceived financial severity was measured with an item adapted from Shmueli [31], with respondents asked to assess on a 5-point Likert scale (1 [insignificant consequences] to 5 [very serious consequences]) the possible financial consequences in the case of COVID-19 infection.

Perceived benefits were measured using 3 items from Chu and Liu [38], with 1 item assessing the perceived individual benefits of COVID-19 vaccines (eg, “COVID-19 vaccines are effective in preventing COVID-19”) and 2 items assessing the perceived community benefits of COVID-19 vaccines (eg, “Having myself vaccinated against COVID-19 is beneficial for the health of others in my community”). Exploratory factor analysis (EFA) indicated that the items represent a single factor, explaining 79.1% of the variance. The Cronbach alpha (α=.95) demonstrated outstanding internal consistency.

Fear was measured with an item from Chu and Liu [38], with respondents asked to assess on a 5-point scale (1 [not at all] to 5 [very much]) how afraid they feel when they think about COVID-19.

Health motivation was measured with 2 items from Shmueli [31]. These items (“I make sure to eat healthy and diverse food every day” and “I ensure that I exercise and work out regularly”) were summed in an index and demonstrated acceptable internal consistency (α=.77; *r*=0.6; *P*<.001; *r_SB_*=0.77).

The HBM also includes susceptibility, perceived barriers, and cues to action, which were measured in our study but only in the subsample of unvaccinated respondents. Because the purpose of this study is to compare different groups of young people, including those who accept vaccination, we did not include these variables in the analysis.

#### TPB Variables

Attitudes toward COVID-19 vaccines were measured using 6 items adapted from Chu and Liu [38]. Respondents were asked to evaluate how they personally feel about COVID-19 vaccination using 5-point scales with various descriptors (negative to positive, undesirable to desirable, bad to good, harmful to beneficial, foolish to wise, and pointless to meaningful). The EFA indicated that the items represent a single factor, explaining 85.7% of the variance, and the Cronbach alpha (α=.97) demonstrated outstanding internal consistency.

Subjective norms were measured using an adapted scale comprising 3 items from Chu and Liu [38]. Respondents were asked to indicate their level of agreement on a 5-point Likert scale (1 [strongly disagree] to 5 [strongly agree]) with statements concerning their similar or significant others (eg, “Most people who are similar to me have been or will be vaccinated against COVID-19”). The EFA showed that the items represent a single factor, explaining 71.5% of the variance, and the Cronbach alpha (α=.88) demonstrated good internal consistency.

To measure self-efficacy, 3 items adapted from the General Self-Efficacy Scale of Schwarzer and Jerusalem [39] were used to ask respondents to rate their agreement on a 5-point scale (1 [strongly disagree] to 5 [strongly agree]) with statements related to managing their health (eg, “If unexpected events related to my health occur, I know how to handle them effectively”). The EFA revealed that the items represent a single factor, explaining 65.3% of the variance. The Cronbach alpha (α=.85) indicated good internal consistency.

#### Confounding Variables

Life satisfaction was measured using a single-item question asking respondents to assess their current satisfaction with life on a scale from 0 (extremely dissatisfied) to 10 (extremely satisfied).

Similarly, trust in the health care system was measured using a single-item question asking respondents to rate their trust in the Slovenian health care system on a scale from 0 (don’t trust at all) to 10 (completely trust).

Trust in science was measured using the Trust in Science and Scientists Inventory [40]. However, since the EFA did not confirm the unidimensionality of the original scale, the measure was slightly adjusted to include 9 items. These items asked respondents to assess their agreement on a 5-point Likert scale (1 [strongly disagree] to 5 [strongly agree]) with statements related to the value of and trust in science (eg, “I believe that the work of scientists contributes to a better life for everyone”). The EFA revealed that the items represent a single factor, explaining 55.1% of the variance, and the Cronbach alpha (α=.92) demonstrated outstanding internal consistency.

Trust in vaccines was measured using 2 items that asked respondents to indicate their agreement on a 5-point Likert scale (1 [strongly disagree] to 5 [strongly agree]) with statements related to the safety and efficiency of COVID-19 vaccines (eg, “I trust that the vaccines available to us against the coronavirus that causes COVID-19 are safe”). The 2 items were summed in an index and demonstrated outstanding internal consistency (α=.92; *r*=0.8; *P*<.001; *r_SB_*=0.92).

Conspiracy theory tendency was measured using 3 items adapted from the Slovenian public opinion research #Novanormalnost [41]. Respondents were asked to assess their agreement with statements suggesting COVID-19 is a part of a conspiracy (eg, “Vaccination against the coronavirus that causes COVID-19 is an attempt to control the population”), using a 5-point Likert scale (1 [strongly disagree] to 5 [strongly agree]). The EFA revealed that the 3 items represent a single factor, explaining 70.3% of the variance, and the Cronbach alpha (α=.87) showed good internal consistency.

Following events related to the pandemic was measured using a single-item question that asked respondents to assess the extent to which they follow pandemic-related events on a 5-point Likert scale (1 [I don’t follow events at all] to 5 [I follow events very often]).

#### Health-Related Variables

Perceived health status was measured using a single-item question on a 5-point Likert scale (1 [very bad] to 5 [excellent]), asking respondents to evaluate their current physical and mental health. The online questionnaire also included a question asking respondents to indicate whether they have a long-term (chronic) condition.

Additionally, single-item questions were used to determine whether respondents have a health or medical professional background and whether they received an influenza vaccine in the past year.

Potential COVID-19 past infection was measured by asking respondents to report their perceived probability of having been infected with COVID-19 in the past (probably infected or probably not infected).

#### Sociodemographic Variables

The online questionnaire included questions asking respondents to indicate their gender and age, with age transformed from a numeric to a categorical variable (15-18 years, 19-22 years, 23-26 years, and 27-30 years) to examine differences across specific age groups. Respondents were also asked to report their current education level (elementary school or less; 2-, 3-, 4-, or 5-year high school; and college, university, or higher education) and their activity status (high school student, student, employed, and unemployed).

Single-item questions were used to determine whether respondents have a partner, consider themselves religious, and live in urban or rural settlements. Respondents were also asked to assess their mother’s or primary carer’s education level (elementary school or less; 2-, 3-, 4-, or 5-year high school; and college, university, or higher education) and their material status on a scale from 1 (significantly below average) to 5 (significantly above average). Due to a low number of observations in categories 1 (significantly below average) and 5 (significantly above average), the material status variable was recoded according to 3 categories: below average, average, and above average.

### Statistical Analysis

Before the analysis, the data were weighted using the random iterative weighting method according to gender, age group, education, and region. The weighting process was conducted by Valicon, the JazVem online panel survey provider. The prevalence of vaccination-related behavior was measured as the percentage of vaccinated respondents, delayers, hesitant respondents, and refusers in the total study sample. A series of EFAs were conducted to determine the internal structure of the selected measurement instruments. Factors were extracted using principal axis factoring with oblimin rotation. We performed EFA using the following criteria: (1) eigenvalue greater than 1, (2) scree test, (3) items loading on the same factor (≥0.04), (4) no cross-loading, and (5) conceptual interpretability of factors. The quality of the measurement instrument was also assessed using reliability analysis, and the Cronbach α coefficient was computed, which ranges between 0 and 1.0. The rough guidelines are that a value of .9 or higher indicates outstanding internal consistency, a value of .8 or higher indicates good internal consistency, a value between .7 and .8 indicates acceptable reliability, and a value between .6 and .7 indicates questionable reliability [42]. For 2-item scales, reliability was estimated using the Spearman-Brown coefficient (*r_SB_*), which was calculated based on the interitem Pearson correlation (*r*). Bivariate analyses were performed to compare groups according to sociodemographic characteristics, health-related variables, confounding variables, HBM variables, and TPB variables, using chi-square tests for categorical variables and ANOVA along with appropriate post hoc tests (Games-Howell) for continuous variables.

Multinomial logistic regression analysis was used to estimate the selected determinants of vaccine uptake with “vaccinated” as the reference group for comparisons. The selection of variables for the multinomial regression model was guided by their statistical significance in bivariate analyses and their theoretical relevance to the research question, ensuring the inclusion of predictors with stronger explanatory power while minimizing the risk of overfitting. Variables that did not show statistically significant associations with the outcome in bivariate analyses were excluded. While the inclusion of all variables was initially considered, challenges with model convergence underscored the importance of a parsimonious model that prioritizes interpretability and robustness. The final model, therefore, balances these considerations to provide reliable and meaningful insights. Confounding variables were included in the multinomial regression model alongside the primary independent variables (HBM and TPB variables, and sociodemographic and health-related variables). By incorporating these confounders into the model, we aimed to account for their potential influence on the outcome variable and to isolate the association between the primary independent variables and the dependent variable. This approach ensures that the estimated effects of the primary predictors are adjusted for the potential bias introduced by the confounding variables. We report adjusted odds ratios (aORs) and 95% CIs. Data were analyzed using IBM SPSS version 27 software.

### Ethical Considerations

Data collection was conducted by the online survey panel provider Valicon, who is a member of the European Society for Opinion and Market Research (ESOMAR), and the data collection and research adhere to the International Chamber of Commerce and ESOMAR international code and professional standards of social research practice. We (the authors) had no access to the respondents’ information and were provided with an anonymized dataset that did not contain any identifiable personal information. The study was conducted in accordance with the Code of Ethics for Researchers of the University of Ljubljana [43] and the World Medical Association’s Declaration of Helsinki on Ethical Principles for Medical Research Involving Human Subjects [44]. The study received approval from the Ethics Committee of the Faculty of Social Sciences, University of Ljubljana (number: 801-2024-025/TD).

## Results

### Participant Characteristics

The results showed that the majority of respondents (232/507, 45.8%) had been vaccinated against COVID-19, 30.0% (152/507) refused vaccination, 12.4% (63/507) were hesitant, and 11.8% (60/507) reported that they intended to be vaccinated against COVID-19 in the near future (ie, delayers). The respondents were on average 23.4 years old (SD 4.4 years), comprising 51.6% (262/507) men and 48.4% (245/507) women (Table 1). The majority had completed high school (290/507, 57.3%), were employed (194/507, 38.3%), were students (184/507, 36.2%), and had a partner (258/507, 50.9%). On average, respondents reported the perceived material status as average (mean 2.1, SD 0.64). Most participants identified as religious (316/507, 62.3%) and resided in urban areas (260/507, 51.3%).

**Table 1:** Sociodemographic participant characteristics in the total sample and according to COVID-19 vaccination–related behavior groups.

Variable			Total sample (N=507), n (%)		Vaccinated individuals (n=232, 45.8%), n (%)		Delayers (n=60, 11.8%), n (%)		Hesitant individuals (n=63, 12.4%), n (%)		Refusers (n=152, 30.0%), n (%)		Chi-square (*df*)			*P* value	
**Gender**														2.8 (3)			.43
	Male	262 (51.6)		127 (48.5)		33 (12.6)		29 (11.1)		73 (27.9)							
	Female	245 (48.4)		105 (42.9)		27 (11.0)		34 (13.9)		79 (32.2)							
**Age (years) groups**														39.7 (9)			<.001
	15-18	102 (20.2)		28 (27.5)		18 (17.6)		17 (16.7)		39 (38.2)							
	19-22	123 (24.3)		66 (53.7)		18 (14.6)		14 (11.4)		25 (20.3)							
	23-26	133 (26.3)		81 (60.9)		6 (4.5)		11 (8.3)		35 (26,3)							
	27-30	148 (29.2)		57 (38.5)		17 (11.7)		20 (13.5)		54 (36.5)							
**Education**														25.6 (6)			<.001
	Elementary school or less	69 (13.6)		15 (21.7)		12 (17.4)		14 (20.3)		28 (40.6)							
	2-, 3-, 4-, or 5-year high school	290 (57.3)		131 (45.2)		35 (12.1)		34 (11.7)		90 (31.0)							
	College, university, or higher education	147 (29.1)		85 (57.8)		12 (8.2)		15 (10.2)		35 (23.8)							
**Activity status**														34.9 (9)			<.001
	High school student	106 (20.9)		31 (29.2)		18 (17.0)		14 (13.2)		43 (40.6)							
	Student	184 (36.2)		109 (59.2)		22 (12.0)		16 (8.7)		37 (20.1)							
	Employed	194 (38.3)		85 (43.8)		16 (8.2)		28 (14.4)		65 (33.5)							
	Unemployed	23 (4.5)		7 (30.4)		4 (17.4)		4 (17.4)		8 (34.8)							
**Partner status**														7.4 (3)			.06
	Do not have a partner	249 (49.1)		109 (43.8)		39 (15.7)		32 (12.9)		69 (27.7)							
	Have a partner	258 (50.9)		123 (47.7)		21 (8.1)		31 (12.0)		83 (32.2)							
**Material status (n=494)**														24.5 (6)			<.001
	Below average	81 (16.4)		26 (32.1)		9 (11.1)		11 (13.6)		35 (43.2)							
	Average	288 (58.3)		123 (42.7)		37 (12.8)		36 (12.5)		92 (31.9)							
	Above average	125 (25.3)		78 (62.4)		8 (6.4)		15 (12.0)		24 (19.2)							
**Mother’s or primary carer’s education (n=488)**														17.4 (6)			.008
	Elementary school or less	40 (8.2)		13 (32.5)		5 (12.5)		6 (15.0)		16 (40.0)							
	2-, 3-, 4-, or 5-year high school	242 (49.4)		99 (40.9)		23 (9.5)		31 (12.8)		89 (36.8)							
	College, university, or higher education	206 (42.2)		112 (54.4)		26 (12.6)		24 (11.7)		44 (21.4)							
**Religiousness**														7.5 (3)			.06
	Nonreligious	191 (37.7)		100 (52.4)		15 (7.9)		22 (11.5)		54 (28.3)							
	Religious	316 (62.3)		132 (41.8)		45 (14.2)		41 (13.0)		98 (31.0)							
**Settlement type**														13.1 (3)			.004
	Urban	260 (51.3)		137 (52.7)		32 (12.3)		28 (10.8)		63 (24.2)							
	Rural	247 (48.7)		95 (38.3)		28 (11.3)		35 (14.1)		90 (36.3)							

The majority of respondents reported having a very good (254/507, 50.2%) or good (150/507, 29.6%) health status (mean 3.7, SD 0.8). Additionally, 67.9% (344/507) of respondents indicated that they were probably not infected by coronavirus in the past (Table 2).

**Table 2:** Health-related participant characteristics in the total sample and according to COVID-19 vaccination–related behavior groups.

Variable			Total sample (N=507), n (%)		Vaccinated individuals (n=232, 45.8%), n (%)		Delayers (n=60, 11.8%), n (%)		Hesitant individuals (n=63, 12.4%), n (%)		Refusers (n=152, 30.0%), n (%)		Chi-square (*df*)			*P* value	
**Perceived health status**														20.9 (12)			.05
	Very bad	6 (1.2)		2 (33.3)		1 (16.7)		0 (0.0)		3 (50.0)							
	Bad	32 (6.3)		18 (56.3)		5 (15.6)		4 (12.5)		5 (15.6)							
	Good	150 (29.6)		63 (42.0)		27 (18.0)		21 (14.0)		39 (26.0)							
	Very good	254 (50.2)		118 (46.5)		20 (7.9)		34 (13.4)		82 (32.3)							
	Excellent	64 (12.6)		30 (46.9)		6 (9.4)		3 (4.7)		25 (39.1)							
**Long-term (chronic) condition**														4.3 (3)			.23
	No	399 (78.7)		181 (45.3)		43 (10.8)		48 (12.0)		128 (32.0)							
	Yes	108 (21.3)		51 (47.2)		17 (15.7)		15 (13.9)		25 (23.1)							
**Potential COVID-19 past infection**														14.4 (3)			.002
	Probably not infected	344 (67.9)		171 (49.7)		46 (13.4)		36 (10.5)		91 (26.5)							
	Probably infected	163 (32.1)		60 (36.8)		14 (8.6)		27 (16.6)		62 (38.0)							
**Health or medical professional background**														5.3 (3)			.15
	No	428 (84.4)		192 (44.9)		46 (10.7)		55 (12.9)		135 (31.5)							
	Yes	79 (15.6)		40 (50.0)		14 (17.5)		8 (10.0)		18 (22.5)							
**Influenza vaccine in the past year**														19.1 (3)			<.001
	No	457 (90.1)		195 (42.7)		55 (12.0)		60 (13.1)		147 (32.2)							
	Yes	50 (9.9)		37 (74.0)		5 (10.0)		3 (6.0)		5 (10.0)							

### Bivariate Analyses and Comparisons Among Vaccination-Related Behavior Groups

Bivariate analyses and comparisons among vaccination-related behavior groups (Table 1) showed that vaccinated individuals were predominantly aged 23 to 26 years (81/133, 60.9%) and were more likely to have a higher education (85/147, 57.8%) and above-average material status (78/125, 62.4%). In comparison, refusers were more common among younger (15-18 years: 39/102, 38.2%) and older age groups (27-30 years: 54/148, 36.5%), had lower levels of education (elementary or less: 28/69, 40.6%), reported below-average material status (35/81, 43.2%), and were more likely from rural areas (90/247, 36.3%; Table 1). Additionally, refusers more often reported a potential COVID-19 past infection (62/163, 38.0%; Table 2). Results also showed that delayers shared characteristics with both vaccinated individuals and refusers, whereas hesitant respondents shared more characteristics with refusers, including rural residence, a lower education level, and a higher rate of potential COVID-19 past infection (Tables 1 and 2).

Table 3 presents participant characteristics across vaccination behavior groups based on confounding, HBM, and TPB variables. Vaccinated individuals reported significantly higher trust in the health care system (mean 6.5, SD 2.2), science (mean 3.9, SD 0.6), and COVID-19 vaccines (mean 3.9, SD 0.9) compared to refusers (Table 3). Conspiracy belief tendencies were on average the highest among refusers (mean 3.6, SD 1.0) and on average the lowest among vaccinated individuals (mean 1.9, SD 1.0). Pandemic-related events were followed more closely by vaccinated individuals (mean 3.1, SD 1.0) and delayers (mean 2.9, SD 0.9) than hesitant individuals (mean 2.7, SD 1.0) and refusers (mean 2.5, SD 1.0). Delayers perceived greater health (mean 2.8, SD 0.9) and financial (mean 2.7, SD 1.1) consequences from COVID-19 compared to refusers. Vaccinated individuals perceived the greatest benefit of vaccination (mean 4.0, SD 0.9), while refusers perceived the lowest benefit (mean 1.8, SD 0.8). Fear of COVID-19 was the lowest among refusers (mean 1.9, SD 1.0) and the highest among delayers (mean 3.0, SD 1.2). According to the TPB model, vaccinated individuals had the highest average scores for positive attitudes toward COVID-19 vaccines (mean 4.1, SD 1.1) and subjective norms (mean 3.8, SD 0.9), which gradually decreased among other groups and were the lowest on average among refusers (Table 2). Self-efficacy in coping with health problems was the highest among vaccinated individuals (mean 3.8, SD 0.7) and refusers (mean 3.7, SD 0.8) but was the lowest among delayers (mean 3.4, SD 0.7). Health motivation did not differ significantly between groups (Table 3).

**Table 3:** Descriptive statistics of confounding variables, health belief model variables, and theory of planned behavior variables in the total sample and according to COVID-19 vaccination–related behavior groups.

Variable			Total sample (N=507), mean (SD)	Vaccinated individuals (n=232, 45.8%), mean (SD)		Delayers (n=60, 11.8%), mean (SD)		Hesitant individuals (n=63, 12.4%), mean (SD)		Refusers (n=152, 30.0%), mean (SD)	ANOVA^a^ * F* (*df*)	*P* value
**Confounding variables**												
	Life satisfaction (scale 0-10)	6.6 (2.2)			6.8 (2.0)		6.1 (2.1)		6.8 (1.9)	6.5 (2.5)	2.1 (3, 502)	.10
	Trust in the health care system (scale 0-10)	5.8 (2.4)			6.5 (2.2)^b^		5.8 (1.6)^b,c^		5.8 (2.4)^d^	4.7 (2.5)^b,c,d^	19.8 (3, 502)	<.001
	Trust in science (scale 1-5)	3.5 (0.8)			3.9 (0.6)^b,c,d^		3.6 (0.6)^b,c^		3.4 (0.67)^b,d^	3.1 (0.8)^b,c,d^	46.1 (3, 502)	<.001
	Trust in vaccines (scale 1-5)	3.0 (1.3)			3.9 (0.9)^b,c,d,e^		3.1 (0.8)^b,c,d,e^		2.5 (0.9)^b,c,d,e^	1.6 (0.8)^b,c,d,e^	226.6 (3, 502)	<.001
	Conspiracy theory tendency (scale 1-5)	2.7 (1.2)			1.9 (1.0)^b,c,d,e^		2.6 (0.8)^b,c,d,e^		3.1 (0.8)^b,c,d,e^	3.6 (1.0)^b,c,d,e^	98.3 (3, 502)	<.001
	Following events related to the pandemic (scale 1-5)	2.9 (1.0)			3.1 (1.0)^b,d^		2.9 (0.9)^c^		2.7 (1.0)^b,d^	2.5 (1.0)^b,c^	12.6 (3, 502)	<.001
**Health belief model variables**												
	Perceived health severity (scale 1-5)	2.3 (1.0)			2.4 (0.9)^b^		2.8 (0.9)^b,c^		2.7 (0.9)^d^	1.9 (0.8)^b,c,d^	23.3 (3, 502)	<.001
	Perceived financial severity (scale 1-5)	2.3 (1.1)			2.4 (1.2)^b^		2.7 (1.1)^c^		2.3 (1.0)	2.0 (1.00)^b,c^	6.1 (3, 502)	<.001
	Perceived benefits (scale 1-5)	3.0 (1.3)			4.0 (0.9)^b,c,d,e^		3.1 (0.7)^b,c,d,e^		2.5 (0.9)^b,c,d,e^	1.8 (0.8)^b,c,d,e^	221.8 (3, 502)	<.001
	Fear (scale 1-5)	2.6 (1.2)			2.8 (1.1)^b^		3.0 (1.2)^c^		2.9 (1.0)^d^	1.9 (1.0)^b,c,d^	31.0 (3, 502)	<.001
	Health motivation (scale 1-5)	3.5 (0.9)			3.5 (0.9)		3.5 (0.9)		3.6 (0.8)	3.6 (1.0)	0.2 (3, 502)	.87
**Theory of planned behavior variables**												
	Attitudes (scale 1-5)	3.0 (1.5)			4.1 (1.1)^b,c,d,e^		3.0 (1.0)^b,c,d,e^		2.4 (0.9)^b,c,d,e^	1.4 (0.7)^b,c,d,e^	260.2 (3, 502)	<.001
	Subjective norms (scale 1-5)	3.0 (1.1)			3.8 (0.9)^b,c,d,e^		3.1 (0.6)^b,c,d,e^		2.5 (0.9)^b,c,d,e^	1.9 (0.8)^b,c,d,e^	159.5 (3, 502)	<.001
	Self-efficacy (scale 1-5)	3.7 (0.7)			3.8 (0.7)^b^		3.4 (0.7)^b,c^		3.6 (0.7)	3.7 (0.8)^c^	4.8 (3, 502)	<.001

^a^Games-Howell post hoc test.

^b^Category has a statistically different mean value (*P*<.05) of the corresponding variable in comparison to the mean value in the other category with the same superscript.

^c^Category has a statistically different mean value (*P*<.05) of the corresponding variable in comparison to the mean value in the other category with the same superscript.

^d^Category has a statistically different mean value (*P*<.05) of the corresponding variable in comparison to the mean value in the other category with the same superscript.

^e^Category has a statistically different mean value (*P*<.05) of the corresponding variable in comparison to the mean value in the other category with the same superscript.

### Predictors of Vaccine Uptake

The results of multinomial logistic regression models with predictors of vaccine uptake, using “vaccinated respondents” as the reference group for comparisons, are shown in Table 4. Variables included in the multinomial regression model were selected based on their statistical significance in bivariate analyses and their theoretical relevance to the research question (more details are provided in the Statistical Analysis subsection in the Methods section). Multinomial regression analysis (Table 4) showed that young adults aged 23 to 26 years (compared with those aged 27 to 30 years) were less likely to delay vaccination (aOR 0.1, 95% CI 0.031-0.5). Young adults who had completed elementary school or less (compared to respondents with higher levels of education) were more likely to delay vaccination (aOR 9.8, 95% CI 1.6-61.1) or were hesitant toward vaccination (aOR 11.7, 95% CI 1.5-89.7). Results also showed that young people who were high school students (compared with unemployed respondents) were less likely to be hesitant (aOR 0.0081, 95% CI 0.0002-0.4; Table 4). Similarly, respondents who were students (aOR 0.1, 95% CI 0.0077-0.9) or employed (aOR 0.1, 95% CI 0.0059-0.6; compared with unemployed respondents) were also less likely to refuse vaccination. Participants whose mothers or primary carers had completed elementary school or less were significantly more likely to refuse vaccination (aOR 6.6, 95% CI 1.3-34.4) compared to those with university-educated mothers or primary carers. Similarly, those whose mothers or primary carers finished high school were more likely to refuse vaccination (aOR 2.8, 95% CI 1.0-8.0) compared to the same university-educated group. The results also showed that respondents who felt that COVID-19 could have greater health consequences for them were more likely to delay vaccination (aOR 2.0, 95% CI 1.2-3.3) or be hesitant toward it (aOR 1.9, 95% CI 1.1-3.2). In addition, more negative attitudes toward COVID-19 vaccines were more likely among delayers, hesitant respondents, and refusers than among vaccinated respondents (Table 4). Compared with vaccinated young people, subjective norms were significantly less likely to be present among hesitant respondents (aOR 0.4, 95% CI 0.2-0.7) and refusers (aOR 0.3, 95% CI 0.2-0.7). Self-efficacy to cope with health problems was less present among respondents who delayed vaccination (aOR 0.5, 95% CI 0.3-0.9) than among vaccinated respondents (Table 4). For the data in Table 4, the R^2^ was 0.7 (Cox and Snell) and 0.8 (Negelkerke), and the model *χ^2^* was 610.2 (*df=*81; *P*<.001).

**Table 4:** Predictors of vaccine uptake by multinomial logistic regression (vaccinated respondents as the reference group).

Predictor variable				Delayers, aOR^a^ (95% CI)		Hesitant individuals, aOR (95% CI)		Refusers, aOR (95% CI)
**Sociodemographic variables**								
	**Age group (reference:** **27-30 years)**							
		15-18 years	2.9 (0.2-39.4)		22.2 (0.8-60.2)		3.13 (0.1-124.9)	
		19-22 years	0.6 (0.12-2.6)		0.8 (0.2-3.6)		0.2 (0.035-1.2)	
		23-26 years	0.1^b^ (0.031-0.5)		0.3 (0.1-1.1)		0.4 (0.1-1.5)	
	**Education (reference:** **college, university, or higher education** **)**							
		Elementary school or less	9.8^c^ (1.6-61.1)		11.7^b^ (1.5-89.7)		6.8 (0.7-65.3)	
		2-, 3-, 4-, or 5-year high school	1.9 (0.7-5.5)		2.5 (0.9-7.4)		4.0^c^ (1.3-12.6)	
	**Activity status (reference: unemployed)**							
		High-school student	0.2 (0.007-4.1)		0.0081^b^ (0.0002-0.4)		0.1 (0.00088-3.0)	
		Student	0.7 (0.1-6.0)		0.1 (0.015-1.2)		0.1^c^ (0.0077-0.9)	
		Employed	0.4 (0.1-3.1)		0.2 (0.023-1.4)		0.1^c^ (0.0059-0.6)	
	**Material status (reference: above average)**							
		Below average	1.8 (0.4-7.5)		1.3 (0.3-5.6)		3.3 (0.7-17.3)	
		Average	1.9 (0.7-5.5)		0.6 (0.2-1.9)		1.3 (0.4-4.4)	
	**Mother’s or primary carer’s education (reference: college, university, or higher education)**							
		Elementary school or less	2.2 (0.5-9.8)		4.2 (0.9-19.7)		6.6^c^ (1.3-34.4)	
		2-, 3-, 4-, or 5-year high school	0.9 (0.4-2.0)		1.4 (0.5-3.5)		2.8^c^ (1.0-8.0)	
	**Settlement type (reference: rural)**							
		Urban	0.9 (0.4-2.0)		0.9 (0.4-2.1)		0.9 (0.3-2.1)	
**Health-related variables**								
	**Potential COVID-19 past infection** **(reference: probably infected)**							
		Probably not infected	1.2 (0.5-2.9)		0.6 (0.2-1.4)		0.9 (0.3-2.6)	
	**Influenza vaccine in the past year** **(reference: yes)**							
		No	1.4 (0.4-5.0)		1.9 (0.4-10.3)		1.5 (0.2-13.0)	
**Cofounding variables**								
	Trust in the health care system			1.1 (0.9-1.4)		1.2 (1.0-1.5)		1.0 (0.8-1.3)
	Trust in science			2.2 (0.9-4.9)		1.2 (0.5-2.6)		2.1 (0.9-5.0)
	Trust in vaccines			0.7 (0.3-1.3)		0.7 (0.4-1.5)		0.5 (0.2-1.1)
	Conspiracy theory tendency			0.9 (0.5-1.6)		1.5 (0.8-2.6)		1.3 (0.7-2.3)
	Following events related to the pandemic			0.9 (0.6-1.4)		0.8 (0.5-1.2)		0.8 (0.5-1.3)
**Health belief model** **variables**								
	Perceived health severity			2.0^b^ (1.2-3.3)		1.9^b^ (1.1-3.2)		0.8 (0.4-1.4)
	Perceived financial severity			1.3 (0.9-1.8)		0.8 (0.5-1.2)		0.8 (0.5-1.3)
	Perceived benefits			0.7 (0.3-1.3)		0.5 (0.2-1.1)		0.6 (0.3-1.4)
	Fear			0.9 (0.6-1.3)		1.2 (0.8-1.8)		0.7 (0.5-1.1)
**Theory of planned behavior** **variables**								
	Attitudes			0.5^c^ (0.3-0.9)		0.5^b^ (0.3-0.9)		0.2^d^ (0.1-0.4)
	Subjective norms			0.7 (0.4-1.3)		0.4^b^ (0.2-0.7)		0.3^d^ (0.2-0.7)
	Self-efficacy			0.5^c^ (0.3-0.9)		0.9 (0.5-1.7)		1.0 (0.5-2.0)

^a^aOR: adjusted odds ratio.

^b^*P*<.01.

^c^*P*<.05.

^d^*P*<.001.

## Discussion

### Principal Findings

The aim of this study was to gain a deeper understanding of the determinants influencing vaccination intention and behavior among young people in Slovenia, an EU country where the adult COVID-19 vaccination rate significantly lagged the EU average. We applied an integrated model to analyze and compare vaccinated and unvaccinated young people, further categorizing the unvaccinated people into 3 groups: those hesitant to vaccinate, those willing but delaying vaccination, and those refusing vaccination against COVID-19. Our goal was to enhance the body of research exploring the decision-making processes within these distinct groups of young people regarding COVID-19 vaccination.

In our study, a substantial proportion of respondents self-reported being vaccinated against COVID-19. This rate was slightly higher than the vaccination rate of the general population (aged 18 years or older) in Slovenia, which was approximately 43% for the first dose by the end of August 2021 [5]. Compared to the vaccinated general population, this is a favorable percentage, as previous studies have indicated that younger adults are among the groups with lower willingness to be vaccinated against COVID-19 [8-15]. This finding may be partially explained by policy interventions in Slovenia during the time of data collection. Notably, the introduction of the so called “COVID-19 pass” (certificate of recovery, vaccination, or testing) was designed to facilitate safer and freer mobility of citizens within the EU. Several studies have shown that the implementation of COVID-19 passes improved both vaccination uptake and intention [45].

Our study demonstrated that unvaccinated individuals are not a homogeneous group. Not all respondents who were unvaccinated at the time of data collection had explicitly refused vaccination against COVID-19. Specifically, almost half of these respondents indicated that they had considered getting vaccinated but had not yet done so. Some of these were hesitant, while others expressed a willingness to be vaccinated but had not yet taken action. Only half of the unvaccinated respondents explicitly refused vaccination against COVID-19.

In comparison to the findings of other studies [46], our study did not find gender to be a significant factor influencing vaccination behavior. However, the influence of other sociodemographic and health-related variables on vaccine uptake among young people aligns with previous research [46]. Key predictors of vaccination were age, education level, activity status, material status, mother’s or primary carer’s education, settlement type, potential COVID-19 past infection, and influenza vaccination in the past year. Vaccinated individuals were predominantly aged 23 to 26 years, were more likely to have higher education, and had above-average material status. In contrast, vaccine refusers were more common among the youngest (15-18 years) and oldest (27-30 years) age groups, had lower education levels (elementary or less), had mothers or primary caregivers with lower education, reported below-average material status, were more likely to reside in rural areas, and more frequently reported a potential past COVID-19 infection. These findings suggest that the factors significantly predicting vaccination behavior are closely related to the primary socioeconomic and cultural resources available to an individual [47]. Delayers, while distinct from both vaccinated individuals and refusers, shared some sociodemographic and health-related characteristics with each group. Hesitant respondents, on the other hand, shared more characteristics with refusers, including rural residence, lower education levels, and higher rates of potential past COVID-19 infection.

The factors from the HBM and TPB models that significantly differentiated the vaccinated respondents from all other groups were greater trust in science, trust in vaccines, trust in the health care system, and positive attitudes toward COVID-19 vaccination. These characteristics appeared to be strongly associated with higher levels of education, as suggested by previous studies [48,49]. Perceived health consequences of COVID-19, negative attitudes toward vaccines, and weaker levels of subjective norms were associated with a greater likelihood of delaying or refusing vaccination. Among unvaccinated respondents, perceived self-efficacy in coping with health problems emerged as a significant factor, particularly for those who were delaying vaccination, as this group reported the lowest levels of self-efficacy. Interestingly, despite exhibiting heightened fear of COVID-19 and perceiving the health and financial burdens of contracting the disease as greater than those reported by other groups, delayers may experience affective and cognitive conflict [25]. This conflict likely stems from the simultaneous presence of fear of contracting the disease and negative attitudes toward COVID-19 vaccines. Combined with low confidence in their ability to manage health issues, this internal conflict may explain their expressed willingness to vaccinate but delay in taking action.

The findings of our study indicated that hesitant respondents shared many similarities with delayers, including the same identified conflict between fear of COVID-19 and negative attitudes toward vaccines. However, hesitant respondents exhibited higher self-efficacy in coping with health problems and reported lower levels of subjective norms. Vaccination refusers, in contrast, reported lower levels of subjective norms, were more likely to have mothers or primary carers with lower levels of education (elementary school or less), and were more likely to hold negative attitudes toward COVID-19 vaccination. Notably, they exhibited a concerning combination of increased self-efficacy in coping with health problems and the highest scores for conspiracy theory tendencies. This profile aligns with a group previously described in the literature as dysfunctionally empowered [50].

### Strengths and Limitations

The strength of this study lies in its comprehensive approach to examining the factors influencing COVID-19 vaccine uptake among young people in Slovenia. It addresses a critical research gap by focusing on the specific population group of young people who have frequently demonstrated hesitant attitudes toward vaccination [13]. By comparing vaccinated and unvaccinated individuals and further categorizing the unvaccinated individuals based on their willingness to be vaccinated, the study provides a detailed analysis of the factors underlying vaccination intention and hesitancy. These insights could inform the development of more targeted and effective public health interventions and strategies to address vaccine hesitancy and improve vaccination rates among the youth.

This study also has some limitations. First, data collection through an online access survey panel may have resulted in overrepresentation of certain demographic groups among young adults who have internet access and are more digitally literate. This could exclude other groups and affect the representativeness of the results. Second, the survey relied on self-reported behavior, which is subject to bias. Third, the cross-sectional design of the study precludes the identification of cause-and-effect relationships. Fourth, variables, such as susceptibility, perceived barriers, and cues to action, from the HBM were measured only in the subsample of unvaccinated respondents. As these variables were not included in the analysis, the study may not fully capture all factors influencing vaccine decision-making across the entire sample. Fifth, the multinomial regression analysis results showed wide CIs, indicating greater uncertainty in the estimated effects of the predictor variables on vaccination-related behavior groups. This may result from small sample sizes, overly complex models, sparse data, or other factors. This should be carefully considered when interpreting our findings. Future research using similar methodological frameworks and a similar design should address these issues to enhance the robustness and interpretability of the results. Finally, the data are limited to the social, cultural, and political contexts of Slovenia, where specific COVID-19–related protective measures and crisis communication may have significantly influenced the vaccination behavior of the youth [25].

### Implications

Our findings suggest that young people exhibit different attitudes toward vaccination against COVID-19. Consequently, public health authorities should implement diverse strategies tailored to address these attitudes and fears, and the underlying factors influencing them. This recommendation extends beyond COVID-19 and is equally relevant to other diseases, particularly those with lower immunization coverage and vaccination rates [51].

According to the stages of change model [37], which provides a framework for understanding how individuals progress toward a desired behavioral change, such as COVID-19 vaccination, we can categorize vaccine refusers as being in the first stage of behavior change, known as precontemplation. Individuals in this stage are often unaware of the problem, are unmotivated to address it, or may even deny its relevance to themselves. Effective strategies for this group include raising awareness of the issue, providing clear information about the benefits of vaccination, and addressing misconceptions with targeted communication tailored to their specific concerns about vaccine safety and health risks. The groups of delayers and hesitant individuals appear to be either in the contemplation or preparation stage. Those in the contemplation stage are considering vaccination but have not yet made a definitive decision. Strategies for this group should focus on helping them weigh the perceived benefits of vaccination, such as protection against COVID-19 and its potential health and financial consequences, against the perceived costs, such as potential vaccine side effects. Delayers, on the other hand, seem to align more with the preparation stage. They are actively planning to get vaccinated but remain the most uncertain group when it comes to making health-related decisions. To support this group, strategies should include encouragement to finalize their decision, such as prompting them to schedule a vaccination appointment and offering reliable information on the effectiveness, safety, and availability of vaccines.

### Conclusions

This cross-sectional study provides survey data on the vaccination behavior (vaccination uptake and refusal) and intention (delayed vaccination and hesitancy) of young people regarding COVID-19 vaccination. It also examines the sociodemographic, health-related, and behavioral predictors of these intentions, integrating constructs from 2 health behavior theories: HBM and TPB.

The findings of our study highlight that young people exhibit diverse behavioral and volitional positions regarding COVID-19 vaccination. Several sociodemographic, health-related, and behavioral factors, such as educational level, material status, subjective norms, perceived self-efficacy, attitudes toward vaccines, fear of contracting the disease, and conspiracy theory tendencies, significantly influence these positions. With regard to vaccination as the desired behavior, individuals are positioned in different stages of behavior change. From a normative perspective, these stages should be carefully considered when designing and implementing measures to promote vaccination for both COVID-19 and other diseases among young people. Greater efforts should be made to target individuals with lower levels of education, those with material disadvantages, and those residing in rural areas.

The findings of this study can guide health policy makers and professionals in optimizing young people’s willingness to vaccinate and can provide valuable insights for addressing other vaccinations, future infectious disease outbreaks, and the implementation of effective preventive measures. Interventions should be tailored to address the primary reasons for nonvaccination, with priority target groups based on their potential for behavior change. Determined refusers may not be the most effective group to focus on initially. Instead, delayers, who demonstrate the greatest willingness to vaccinate among unvaccinated individuals, require targeted strategies, such as those aimed at increasing their self-efficacy in managing health-related decisions.

## Appendix

Multimedia Appendix 1 CHERRIES (Checklist for Reporting Results of Internet E-Surveys).

## Abbreviations

aOR: adjusted odds ratio
EFA: exploratory factor analysis
ESOMAR: European Society for Opinion and Market Research
EU: European Union
HBM: health belief model
TPB: theory of planned behavior

## Data Availability

The data underlying this article will be shared by the corresponding author upon reasonable request.
