# Supplementary material for: Predictors of COVID-19 Vaccination Intention and Behavior Among Young People in a European Union Country With Low COVID-19 Vaccination Rates: Cross-Sectional Study
Source: JMIR Public Health Surveill. 2025 Feb 21;11:e64653. doi: 10.2196/64653 (PMC11890135; doi:10.2196/64653)
Supplement: Multimedia Appendix 1 [file publichealth_v11i1e64653_app1.pdf]

Multimedia Appendix 1

CHERRIES (Checklist for Reporting Results of Internet E-Surveys)

Item Category	Checklist item	Explanation
**Design**	Target population	Young people in Slovenia in the age group 15-30 years recruited via online access survey panel JazVem provided by Valicon, Slovenian marketing research company. Quotas were used to achieve a sample quasi-representative of the general population in Slovenia by age, gender, education, and region based on data from Statistical Office of the Republic of Slovenia.
	Survey topics	Perception of COVID-19-related protective measures; vaccination against COVID-19 behavior; questions related to HBM and TPB constructs; trust in science and vaccine; respondent's health and sociodemographic questions.
**IRB approval and informed consent process**	IRB approval	Research was conducted in line with the Code of Ethics for Researchers of the University of Ljubljana and the World Medical Association's Declaration of Helsinki on Ethical Principles for Medical Research Involving Human Subjects. The study received consent from Ethical Committee of Faculty of Social Sciences, University of Ljubljana (No. 801-2024-025/TD).
	Informed consent	Members voluntarily sign up for the JazVem online access survey panel, agreeing to the panel's terms and privacy conditions, signing a participation consent form, providing baseline information, and regularly receiving emails inviting them to participate in various

Item Category	Checklist item	Explanation
		research studies. Respondents selected through quota sampling received an email invitation. By clicking the survey link in the email, potential respondents were directed to the survey's introduction page, which included information about the research purpose, the survey's length, details about the investigator and their contact information, as well as a statement that participation was voluntary and that aggregated results might be published.
	Data protection	Valicon and its JazVem online access survey panel are reputable web services that handle all personal information, including email addresses, in compliance with national and EU laws and safeguard data using standard security procedures. The online survey panel provider Valicon is also a member of the European Society for Opinion and Market Research (ESOMAR), ensuring that their data collection and research practices adhere to the International Chamber of Commerce (ICC) and ESOMAR international code, as well as professional standards for social research. The authors had no access to respondents' personal information and were provided with an anonymized dataset containing no identifiable personal data.
**Development and pre-testing**	Development and testing	The survey questionnaire, consisting of 79 questions in Slovene, was developed by the authors. Its content validity was assessed by

Item Category	Checklist item	Explanation
		eight experts from various fields, including public health, health communication, sociology, communication studies, statistics, social science methodology, and psychology.
**Recruitment process and description of the sample having access to the questionnaire**	Open survey versus closed survey	The survey was open to participants registered with the JazVem online survey panel who used a valid email address and received an email invitation to participate.
	Contact mode	Valicon, the online survey panel provider, invited potential respondents to participate through its JazVem online survey panel system.
	Advertising the survey	Respondents (panelists of the JazVem online access survey panel) selected through quota sampling received an email invitation. The invitation was designed in accordance with the latest guidelines for designing email invitations for online surveys.
**Survey administration**	Web/E-mail	The survey was administered by Valicon, using its JazVem online survey panel system. Potential respondents were selected with quota sampling and invited via email.
	Context	The email invitation contained only information about the online survey.
	Mandatory/voluntary	Participation in the online survey was voluntary.
	Incentives	JazVem survey panelists receive small incentives in the form of points for completing surveys, which can be redeemed for modest rewards. The number of points offered and assigned is determined separately for each survey, in accordance with Valicon's internal policy.

**Item Category**	**Checklist item**	**Explanation**
	Time/Date	The survey was administered and available from August 11th to August 17th, 2021.
	Randomization of items or questionnaires	No sets of items within the item tables were randomized, and variables were not randomized to preserve the logical structure of the questionnaire.
	Adaptive questioning	Where relevant, conditioning and routing were used.
	Number of items	In general, the survey was structured with one measurement instrument per page. However, when a measurement instrument contained more than 10 items, the table was split across additional pages. The maximum number of items per respondent was 237, and completing the survey took approximately 15–20 minutes.
	Number of screens (pages)	The total number of pages visible to participants was 71, but due to skip patterns, the actual number of pages viewed was lower.
	Completeness check	A completeness check was performed during the questionnaire administration. Responses were required to proceed to subsequent questions, except for routing questions.
	Review step	Given the survey's length, participants were not required to review their responses upon completion. A 'back' button was available for those who wished to edit previous answers.
**Response rates**	Unique site visitor	Each JazVem panel member is assigned a unique ID and can complete only one survey questionnaire per project. Individuals without a pre-assigned ID (non-panel members) cannot access the survey. Panel members are permitted to have only one

Item Category	Checklist item	Explanation
		ID linked to their email address. If the panel provider discovers that a member has registered multiple profiles with different email addresses, the duplicate accounts are blocked, and the member is removed from the panel, as this violates the participation terms.
	View rate (Ratio of unique survey visitors/unique site visitors)	It was not possible to calculate this.
	Participation rate (Ration of unique visitors who agreed to participate/unique first survey page visitors)	47.5% (568/1197)
	Completion rate (Ration of users who finished the survey/users who agreed to participate)	92.4% (525/568)
**Preventing multiple entries from the same individual**	Cookies used	Respondents were informed about cookies in line with the EU cookie policy.
	IP check	IP addresses were not collected from participants, but the online panel survey system JazVem collects them and prevents multiple entries from the same IPs. Each JazVem panel member is assigned a unique ID and is permitted to complete only one survey questionnaire per project.
	Log file analysis	Log file analysis was not performed.
	Registration	To participate in the online survey, respondents were required to be registered with the JazVem online access survey panel.
**Analysis**	Handling incomplete questionnaires	There were no incomplete questionnaires.
	Questionnaires submitted with an atypical timestamp	Data from respondents who finished the survey questionnaire in less than five minutes were eliminated from the data set.
	Statistical correction	Before the analyses, the data were weighted using the random iterative weighting

Item Category	Checklist item	Explanation
		method based on gender, age group, education, and region. The weighting process was conducted by Valicon, the JazVem online panel survey provider.
